# Microbial community potentially responsible for acid and metal release from an Ostrobothnian acid sulfate soil

**DOI:** 10.1111/1574-6941.12084

**Published:** 2013-02-26

**Authors:** Xiaofen Wu, Zhen Lim Wong, Pekka Sten, Sten Engblom, Peter Österholm, Mark Dopson, Cindy Nakatsu

**Affiliations:** 1 Centre for Ecology and Evolution in Microbial Model Systems (EEMiS), Biology and Environmental Sciences, Linnaeus UniversityKalmar, Sweden; 2 Vaasa University of Applied SciencesVaasa, Finland; 3 Novia University of Applied SciencesVaasa, Finland; 4 Department of Geology and Mineralogy, Åbo Akademi UniversityÅbo, Finland

**Keywords:** Pyrite, metastable iron sulfide, acidification, molecular phylogeny, acidophile

## Abstract

Soils containing an approximately equal mixture of metastable iron sulfides and pyrite occur in the boreal Ostrobothnian coastal region of Finland, termed ‘potential acid sulfate soil materials’. If the iron sulfides are exposed to air, oxidation reactions result in acid and metal release to the environment that can cause severe damage. Despite that acidophilic microorganisms catalyze acid and metal release from sulfide minerals, the microbiology of acid sulfate soil (ASS) materials has been neglected. The molecular phylogeny of a depth profile through the plough and oxidized ASS layers identified several known acidophilic microorganisms and environmental clones previously identified from acid- and metal-contaminated environments. In addition, several of the 16S rRNA gene sequences were more similar to sequences previously identified from cold environments. Leaching of the metastable iron sulfides and pyrite with an ASS microbial enrichment culture incubated at low pH accelerated metal release, suggesting microorganisms capable of catalyzing metal sulfide oxidation were present. The 16S rRNA gene analysis showed the presence of species similar to *Acidocella* sp. and other clones identified from acid mine environments. These data support that acid and metal release from ASSs was catalyzed by indigenous microorganisms adapted to low pH.

## Introduction

Metal sulfide, such as pyrite (FeS_2_), containing anoxic sediments (Rickard & Luther, 2007) are found in several coastal regions such as Australia and the Baltic (as well as some inland areas) and are termed ‘potential acid sulfate soil (PASS) materials’. In the Baltic Sea, these sediments also commonly contain metastable iron sulfide (FeS_*n*_; *n* = 1.0–1.3). When the iron sulfides present in PASSs are exposed to atmospheric oxygen, such as via drainage for agricultural purposes, oxidation reactions can mobilize acidity and metals (Burton *et al*., 2006). At this point, they are termed ‘acid sulfate soil (ASS) materials’. Oxidation of the metal sulfides can have a major effect on the iron and sulfur cycles and cause significant environmental damage (Cook *et al*., 2000). The environmental pressures caused by ASS are increasing due to drainage for use in agriculture, forestry, residential housing or industry. The largest areas of ASS in Europe occur in Finland where acid run-off has caused large-scale fish kills as recently as 2006.

It is generally accepted that metastable iron sulfide weathering in PASS is abiotically, rapidly oxidized to form Fe^3+^ and elemental sulfur (S^0^), as well as other inorganic sulfur compounds (ISCs; Ward *et al*., 2004). The subsequent oxidation of sulfur (Burton *et al*., 2009) and microbial-catalyzed FeS_2_ dissolution may be mediated by the indigenous microorganisms and is termed ‘leaching’ (Schippers & Sand, 1999). However, Burton *et al*., (2009) showed that at near neutral pH, the unidentified microorganisms were in competition with rapid abiotic oxidation. The ultimate products of the iron sulfide oxidation are Fe^3+^-oxyhydroxides, protons, and sulfate (Rickard & Luther, 2007).

The role of acidophilic microorganisms (pH optimum ≤ 5) in the formation of acidic, metal laden solutions by catalyzing sulfide mineral leaching is well documented (reviewed in Rohwerder *et al*., 2003). Despite this, knowledge of the microbial populations in ASS is extremely limited. *Acidithiobacillus ferrooxidans* and *Acidithiobacillus thiooxidans* have been isolated from ASS although they were not attributed to contribute to the initial pH decrease to pH 4 (Arkesteyn, 1980). In addition, cell numbers of acidophilic acidithiobacilli in the River Sirppujoki, Finland, correlated with acid discharge, suggesting the microorganisms played a role in PASS oxidation (Niemelä & Tuovinen, 1972). Two Australian PASS and ASS plate isolates had 16S rRNA gene sequences that align with the Fe^2+^-oxidizing acidophiles *Alicyclobacillus* spp. and *Thiomonas* spp. (E. Watkin, pers. commun.), while molecular phylogenetic methods identified a further 40 operational taxonomic units (OTUs). A Japanese PASS also contains *A. thiooxidans* (Ohba & Owa, 2005). However, experience from low-temperature sulfide mineral environments and acid mine drainage (AMD) has demonstrated a significantly simpler mixed population than many soils (Dopson *et al*., 2007). Also, only a single acidophile capable of low-temperature growth on Fe^2+^ and/or ISCs has been reported (Dopson *et al*., 2007; Kupka *et al*., 2007, 2009).

This study reports physical and chemical characteristics in the soil from the Risöfladan experimental field, Finland; identifies the molecular phylogeny of boreal PASS and ASS direct from the environment as well as after enrichment at low pH; and finally reports accelerated acid and metal release from PASS in the presence of microorganisms enriched from the ASS. The identified microbial species were connected with their potential role in acid and metal release and subsequent environmental pollution.

## Materials and methods

### Site description and sampling

**Fig. 1:**
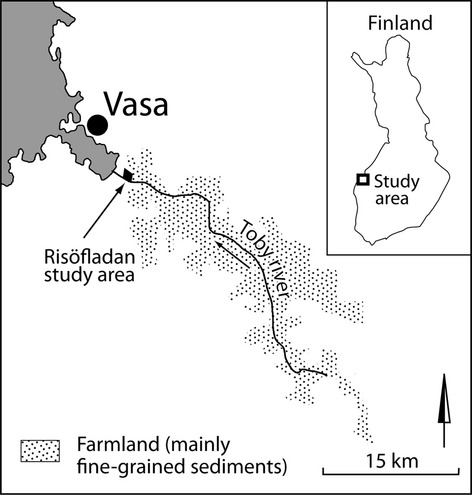
Map of the Risöfladan study site and the local catchment area.

PASS and ASS samples were collected from the Risöfladan experimental field, Finland, that had been drained by embankment and used as agricultural land for more than 40 years (Fig. 1 and Supporting Information, Fig. S1). The parent sediment is made up of *c*. 50% metastable iron sulfides (FeS_*n*_; *n* = 1.0–1.3) and 50% FeS_2_. The study area has been extensively described (Astrom *et al*., 2007; Boman *et al*., 2010).

A soil/sediment profile was sampled with an auger, and pH was measured in the field at vertical depth intervals of 20 cm from the surface down to a depth of 3 m. Deionized water was added in an *c*. 1 : 1 ratio with the soil surface such that the electrode (Mettler InLab Surface electrode with a flat surface that can be used directly on soil material) may be inserted a few millimeters into the soil sample to allow proper contact between the electrode and soil (e.g. Österholm & Åström, 2002). Soil for direct molecular phylogenetic analysis was aseptically sampled using a sterile spatula and immediately sealed in sterile plastic bags. Samples for enrichment cultures were kept moist at 4 °C while samples for DNA extraction were frozen within 2 h of sampling. The soils were sampled from the following depths: metal sulfide–free plough layer (30 cm below the surface); the red oxidized, acidic layer (75 cm); the mixed partially oxidized pH 4–6 layer (127 cm); and the dark reduced zone (> 180 cm).

Fresh samples for the enrichment and leaching experiments were taken from: (1) the upper oxidized zone (depth 40 cm) as an inoculum to enrich for acidophilic microorganisms and for leaching experiments; (2) from the unoxidized soil (depth 3 m); and (3) a mixture of the partially oxidized soils (depth 130 cm) present at the oxidation front.

### Enrichment cultures and catalysis of metal release from PASS

Partially oxidized soil (*c*. 5 g) was inoculated into Erlenmeyer flasks containing 100 mL mineral salts medium (MSM without trace elements) at pH 3.0 (Dopson & Lindström, 1999). In addition to the Fe^2+^ and S^0^ contained in the PASS, additional growth substrates of either 50 mM Fe^2+^, 5 mM tetrathionate, or 0.02% (wt/vol) yeast extract were added to enrich for autotrophic Fe^2+^ oxidizers, autotrophic ISC oxidizers, and heterotrophic acidophilic microorganisms, respectively. The MSM was autoclaved before filter-sterilized (Millipore 0.2 μm cellulose acetate filter) Fe^2+^ or tetrathionate or autoclaved yeast extract was added as required. Cultures were shaken at room temperature (incubation time 57 days), and cell growth confirmed by qualitative observation of an increase in cell numbers in the liquid culture under a Zeiss Primo Star iLED microscope.

Metal and acidity release from PASS was investigated using 1.5-L working volume stirred tank reactors (STRs) containing MilliQ ultrapure water, 10% (wt/vol) soil material, and a temperature of 22 ± 1 °C. The STRs were stirred with an impellor at 150 r.p.m. and aerated with 300 mL min^−1^ air injected just above the impellor. Five separate leaching experiments were carried out: (1) the unoxidized fraction (from a depth of 300 cm) without additional inoculation at circumneutral pH; (2) the partially oxidized soil fraction (depth 130 cm) without additional inoculation or pH control; (3) the partially oxidized soil fraction without enriched microorganisms plus the microbial inhibitor thymol (0.08% wt/vol) at an initial pH of 4.2; (4) the partially oxidized soil fraction without enriched microorganisms (only indigenous microorganisms) at an initial pH of 4.2; and (5) the partially oxidized soil fraction using the enriched acidophilic microorganisms and an initial pH of 4.2. The partially oxidized soil fraction (experiment 5) was inoculated with equal culture volumes from the acidophile enrichment cultures grown on Fe^2+^, tetrathionate, and yeast extract (described earlier). Analyses included total leached Fe (Fe_tot_) and soluble leached Fe (Fe_sup_), soluble Fe^2+^, pH, and redox potential. Fe_tot_ was prepared by acid digesting homogenous samples of PASS slurry from the STRs that included soluble Fe^2+^ and Fe^3+^ as well as secondary iron precipitates produced as a result of sulfide mineral oxidation that were redissolved by incubation with 5 M HCl, but not the iron in the unoxidized pyrite (the HCl treatment does not solubilize iron from the pyrite lattice). Fe_sup_ was a measure of soluble Fe^2+^ and Fe^3+^ after centrifugation to remove PASS particles followed by incubation with 5 M HCl. The extracted Fe_tot_ and Fe_sup_ were measured by atomic adsorption spectrometry (Dopson & Lindström, 1999). Soluble Fe^2+^ was measured by titration of the centrifuged sample with ceric sulfate (Dopson & Lindström, 1999). Redox potentials against the standard hydrogen electrode (SHE) were calculated from the measured redox potentials against the Ag^0^-AgCl (3.5 M KCl) as previously described in Dopson & Lindström (1999) by addition of 205 mV. Finally, 10 mL samples were removed and centrifuged at 10 000 ***g*** for 10 min, and the pellet was frozen for molecular phylogenetic analysis. Leaching experiments and controls were carried out as at least triplicates except leaching of the partially oxidized soil fraction inoculated with the low-pH enrichment cultures that was carried out as a duplicate. Data are presented as averages ± SDs.

### Molecular phylogenetic analysis

PASS and ASS samples (5 g) were suspended in 10 mL of 100 mM Tris containing 10 mM EDTA (TE; pH 8) to adjust the culture pH before DNA extraction and to remove divalent metals by complexing with EDTA. The cells were collected by centrifuging 1 mL at 10 000 ***g*** for 10 min, washed twice, and resuspended in 1 mL TE. Lysozyme (10 mg mL^−1^) was added and incubated at 37 °C for 2 h before cells were lysed by bead beating for 3 min (Dopson & Lindström, 2004; Morales *et al*., 2005). DNA was isolated using the Wizard Genomic DNA purification kit (Promega) according to the manufacturer's instructions. Partial 16S rRNA gene fragments were amplified utilizing illustra PuReTaq Ready-To-Go PCR Beads (GE Healthcare) on an Applied Biosystems 2720 Thermal Cycler (Morales *et al*., 2005). The bacterial PCR primers were GM5F (CCTACGGGAGGCAGCAG) and 907R (CCCCGTCAATTCCTTTGAGTTT; Morales *et al*., 2005) and the archaeal primers ARC344f (ACG GGG CGC AGC AGG CGC GA) and ARC915R (GTG CTC CCC CGC CAA TTC CT).

PCR products were cloned into the pGEM-T Easy Vector System (Promega) and transformed into *Escherichia coli*. The transformants were spread onto Luria–Bertani (LB) plates, which were supplemented with 5-bromo-4-chloro-indolyl-β-d-galactopyranoside (60 μg mL^−1^), isopropyl β-d-1-thiogalactopyranoside (238.3 μg mL^−1^), and ampicillin (100 μg mL^−1^) and incubated overnight at 37 °C. Clones were cultivated in LB medium containing ampicillin (100 μg mL^−1^), and the plasmid DNA was purified using QIA prep Spin Miniprep kit (Qiagen; Dopson & Lindström, 2004). The purified plasmid DNA was digested with two restriction enzymes (MspI and HhaI). OTUs were identified by restriction fragment length polymorphism (RFLP) patterns. Representative clones were sequenced by Macrogen, and the chimeric sequences were checked and removed using DECIPHER (Wright *et al*., 2012). The sequences were compared with the GenBank database using blast before phylogenetic trees were constructed by neighbor joining using Molecular Evolutionary Genetics Analysis version 4.0 (Tamura *et al*., 2007). Named reference species were used in the phylogenetic trees and Table S1 when present in the top 100 blast hits; otherwise, the most similar uncultured clone was named. 16S rRNA gene sequences were submitted to GenBank with the accession numbers: JX869406–JX896486 and A10G12 (Table S1).

## Results and discussion

### Physicochemical characteristics of the soil and parent sediment

The plough layer (0–0.4 m below the surface; depth profiles are indicated in Fig. S1) had a high organic matter content and a pH > 4.7 due to extensive surface liming. In the ‘oxidized’ zone (0.4–1.2 m), the soil (Munsell color observed in field: 2.5Y 4/2) had a well-developed structure with an abundance of iron oxide coatings (5YR 5/5; Fig. S1) on aggregates/cracks. The pH was between 3.7 and 4.2 to a depth of 1.2 m. In the semi-oxidized ‘transition’ zone (1.2–1.8 m; Gley1 4/10Y), pH increased rapidly with depth and was circumneutral in the ‘un-oxidized’ parent sediment below 1.8 m. Iron oxides were rather scarce in the transition zone occurring in vertical near-hexagonal-shaped cracks. The unoxidized parent sediment was blackish (Gley2 2.5/5PB; Fig. S1), typical for metastable iron sulfides without structure or iron oxides (Boman *et al*., 2008).

Observations with a scanning electron microscope equipped with an energy-dispersive X-ray analyzer (EDXA) showed that sulfides also occurred abundantly as framboidal pyrite (Fig. S2) with a Fe/S ratio near 1 : 2. According to a study by Nordmyr *et al*. (2006) who studied five sites in the Ostrobothnian coastal area, the total sulfur concentration in the parent sediment and transition zone is between 0.8% and 1.2% consisting almost exclusively of sulfides, while the oxidized (leached) zone had sulfur concentrations between 0.2% and 0.4% with significant amounts of sulfides (approximately half of total S) and sulfate. Boman *et al*. (2008), who studied one site in the Ostrobothnian coastal area, found that up to half of the sulfides in the parent sediment consist of metastable iron sulfides, while only pyrite sulfide (more resistant) can be found in the oxidized zone. Below the plough layer, the clay and organic matter content in the mineral soil is 30–40% (hydrometer reading) and 4–6% (loss on ignition), respectively. From the data in this and previous studies, it can be concluded that the physicochemical characteristics of the soil and parent sediment are similar to soils in a larger area covering several catchments in the Vaasa region (Österholm & Åström, 2002; Nordmyr *et al*., 2006; Boman *et al*., 2008). Thus, the physicochemical conditions for the microorganisms are likely to be similar in the study area.

From 22 June 2011 (monitoring started), the temperature at 60 cm was relatively stable at *c*. 14 °C until the beginning of September after which it started to decrease and was 4 °C on 14 December (end of monitoring period; Fig. S3). During the same period, the temperature at 150 cm increased rapidly from 6 to 12 °C at the end of August, started to decrease again in the beginning of October, and was 8 °C in December. These values were sufficiently high for psychrotolerant and/or psychrophilic microorganisms to be active, such as the Fe^2+^- and ISC-oxidizing acidophile *Acidithiobacillus ferrivorans* that grows as low as 4 °C (Kupka *et al*., 2007, 2009).

### Molecular phylogeny of environmental PASS and ASS samples

No 16S rRNA gene sequences were amplified with the archaeal primers in any of the analyses suggesting that they did not constitute a major portion of the population and the results will not be discussed further. Phylogenetic analysis of the microorganisms present in the plough and oxidized layers (V1 and V2) identified 16S rRNA gene clones with sequence similarity to known acidophiles and clones previously identified from acidic, metal- and sulfur-containing environments (Table S1 and Figs S4–S7). These included clone V1A12 that was 99% similar to *A*. *ferrooxidans*, a Fe^2+^- and ISC-oxidizing acidophile often identified from acidic metal sulfide environments (Dopson & Johnson, 2012); V1G9 that was 98% similar to an uncultured *Acidimicrobiaceae* clone isolated from an extreme acid environment (Sanchez-Andrea *et al*., 2011); V2B5 that was 99% similar to the uncultured bacterium clone O218406E05 from the acid- and metal-contaminated Rio Tinto river, Spain (Amaral-Zettler *et al*., 2011); and V2C2 and V2C4 that were both 99% similar to a clone identified from AMD (NCBI direct submission). Another represented type of environment was the cold, for example, clone V2A5 was 99% similar to a clone isolated from arctic soil (Borin *et al*., 2010) and V1P14 was 98% similar to a clone identified from a glacier (Pradhan *et al*., 2010). In total, 54% of sequenced clones from the plough and oxidized layers were most similar to known acidophiles or sequences identified in acidic environments, suggesting that these microorganisms were also present in ASS. The microorganisms present in the deeper, partially oxidized, and reduced layers contained several clones previously isolated from anaerobic environments. These included clones V3C6 and V3D5 that were most similar to microorganisms identified from anaerobic wastewater treatment plants (NCBI direct submissions) and V4H2 that was 99% similar to an anoxic fjord sediment (NCBI direct submission).

### Metal and acid release from PASS

Leaching of the unoxidized fraction without pH control or additional inoculation (leaching experiment 1) resulted in a pH decrease from 7.9 ± 0.1 to 3.0 ± 0.4 (Table 1), potentially due to abiotic oxidation of the metastable iron sulfides as a result of aeration, acid generating precipitation of Fe^3+^, and microbial ISC oxidation to sulfuric acid (Dopson & Lindström, 1999). During the same time period, the total iron (Fe_tot_) increased from 22.4 ± 0.8 mM at day 0 to 30.7 ± 1.5 mM after 45 days (Table 1). The soluble Fe^2+^ concentration increased from 3.9 ± 0.1 to 5.8 ± 0.4 mM after 20 days (data not shown) and subsequently decreased while the redox potential (vs. SHE) reached 846 ± 3 mV after 45 days (Table 1). Leaching the partially oxidized soil fraction without pH control or enriched microorganisms (leaching experiment 2) had an initial pH of 4.2 ± 0.1 due to previous inorganic metastable iron sulfide oxidation and the potential action of Fe- and ISC-oxidizing microorganisms. During leaching, the pH decreased to 3.4 ± 0.1 after 20 days (data not shown) and remained stable for the remainder of the experiment (Table 1). Over the same time, both the redox potential and Fe_tot_ increased while the soluble Fe^2+^ decreased from 3.3 ± 0.3 to 2.7 ± 0.3, suggesting some Fe^2+^ oxidation had occurred. The release of metal and acidity coupled to a decrease in soluble Fe^2+^ supports that PASS contain acid-tolerant/acidophilic microorganisms capable of Fe^2+^ and ISC oxidation.

**Table 1:** Leaching of the unoxidized soil fraction without pH control [i.e. near neutral pH; number of replicates (*n*) = 3], the partially oxidized soil fraction without pH control (*n* = 3), and the partially oxidized soil fraction set at an initial pH of 3 including controls (*n* = 2–5). Data are presented as averages ± SD

	Inoculum	Days*	Fe_tot_ (mM)	Fe_sup_ (mM)	Fe^2+^_sol_ (mM)	Redox (mV)†	Redox vs. SHE (mV)‡	pH
1. Unoxidized§	None	0	22.4 ± 0.8	0.0 ± 0.0	3.9 ± 0.1	445 ± 1	650 ± 1	7.9 ± 0.1
		45	30.7 ± 1.5	0.2 ± 0.0	4.6 ± 0.3	641 ± 3	846 ± 3	3.0 ± 0.4
2. Partially oxidized	None	0	16.5 ± 0.4	0.4 ± 0.2	3.3 ± 0.3	399 ± 15	604 ± 15	4.2 ± 0.1
		45	25.2 ± 2.4	0.0 ± 0.0	2.7 ± 0.3	590 ± 5	795 ± 5	3.5 ± 0.1
3. Partially oxidized	None (plus thymol)	0	18.6 ± 4.4	0.2 ± 0.1	NA¶	265 ± 10	470 ± 10	4.2 ± 0.1
		8	23.9 ± 3.4	0.1 ± 0.1	NA	312 ± 4	517 ± 4	4.2 ± 0.1
4. Partially oxidized	None (no thymol)	0	21.9 ± 1.9	0.1 ± 0.0	0.0 ± 0.0	174 ± 20	379 ± 20	4.2 ± 0.1
		8	26.6 ± 3.5	0.5 ± 0.3	0.0 ± 0.0	461 ± 39	666 ± 39	3.0 ± 0.2
5. Partially oxidized	Enrichment	0	27.1 ± 2.7	0.4 ± 0.0	2.2 ± 1.9	327 ± 4	532 ± 4	4.2 ± 0.1
		8	39.1 ± 3.6	0.4 ± 0.0	1.2 ± 1.6	582 ± 6	787 ± 6	3.0 ± 0.1

* Number of days leaching experiment was carried out.

† Pt electrode against Ag^0^/AgCl reference with 3.5 M KCl.

‡ Redox potentials vs. the SHE were calculated from the measured redox potential values by the addition of 205 mV.

§ Numbers denote the experiment numbers listed in the methods and results.

¶ Data not available as thymol interferes with the ferrous iron assay.

**Fig. 2:**
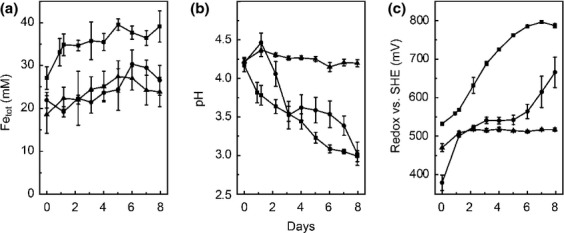
Leaching of the partially oxidized soil fraction at an initial pH of 4.2 showing total iron (Fe_tot_; a), pH (b), and redox potential (vs. SHE) (c). Symbols: enrichment cultures of acidophilic microorganisms (▪); no added microorganisms such that there are only indigenous microorganisms present (•); and no added microorganisms plus the bacterial inhibitor thymol (▲). Data points are averages of replicates (*n* = 2–5) ± SD.

Leaching of the metastable iron sulfides and pyrite without additional acid-tolerant/acidophilic enrichment cultures and in the presence of the inhibitor thymol (leaching experiment 3) had a significantly lower initial redox potential than that for the other two leaching experiments (Fig. 2c). The most likely explanation for this was that the soil material was partly disturbed and contained cracks that transport air, creating large gradients in the soil material redox potential. As a result of aeration with 300 mL min^−1^ air, the redox potential initially increased causing rapid abiotic oxidation of the metastable iron sulfide and a small increase in Fe_tot_ was observed. However, due to the lack of microbial activity, no further increase in the redox or Fe_tot_ was observed and the pH did not decrease (Fig. 2). In the leaching without inoculation with acid-tolerant/acidophilic enrichment cultures (leaching experiment 4), the Fe_tot_ concentrations at the 0- and 8-day time points were not significantly different from the control with thymol (one-way anova at 0.05 confidence = 0.22 and 0.68, respectively). However, the presence of indigenous microorganisms resulted in a decreased pH and increased redox potential (Fig. 2). Finally, addition of acid-tolerant/acidophilic enrichment cultures (leaching experiment 5) resulted in a significant increase in Fe_tot_ after 8 days compared to leaching with and without thymol (anova value = 0.02). The presence of enriched acid-tolerant/acidophilic microorganisms also resulted in a more rapid pH decrease and a higher final redox potential (Fig. 2 and Table 1). In addition, the soluble Fe^2+^ concentration initially increased to 2.5 ± 1.8 mM after 2 days (data not shown) and subsequently decreased to 1.2 ± 1.6 mM after 8 days (Table 1), suggesting that Fe^2+^-oxidizing microorganisms were active (Dopson & Lindström, 2004). These data are typical of previous studies of metal dissolution from sulfide minerals (Dopson & Lindström, 2004) and support that indigenous acid-tolerant/acidophilic microorganisms in the PASS/ASS catalyze metal and acid release.

### Molecular phylogeny after enrichment at low pH

Acidophilic microorganisms were enriched at pH 3 on Fe^2+^, the ISC tetrathionate, and yeast extract-containing media to identify acid-tolerant and/or acidophilic species (Table S1 and Fig. S8). Clones A4E9 and A4F2 were 100 and 99% similar to the named acidophile species *A. ferrivorans* and *A. ferrooxidans*, respectively (reviewed in Dopson & Johnson, 2012). *A. ferrivorans* is a psychrotolerant Fe^2+^-oxidizing acidophile (Dopson *et al*., 2007; Kupka *et al*., 2007) and ISC-oxidizing acidophile (Kupka *et al*., 2009; Liljeqvist *et al*., 2011) previously identified in low-temperature, acidic sulfide mineral mining environments (Hallberg *et al*., 2010). Other clones from the enrichment cultures similar to acidophiles included A4E6 that was 97% similar to a *Sulfobacillus* sp., that is, a Fe^2+^- and ISC-oxidizing facultative chemothithotroph as well as C4B6 and C4C6 that were 99% similar to *Thiomonas* sp. FB-Cd involved in iron cycling in a heavy-metal-contaminated, slightly acidic creek sediment (NCBI direct submission). In addition, a further 45 sequences were most similar to environmental clones identified from acid mine environments containing, or are affected by, sulfide minerals including from Rio Tinto, Spain; an arsenic rich AMD in Carnoules, France; and the La Zarza-Perrunal mine in the Iberian Pyrite Belt.

**Fig. 3:**
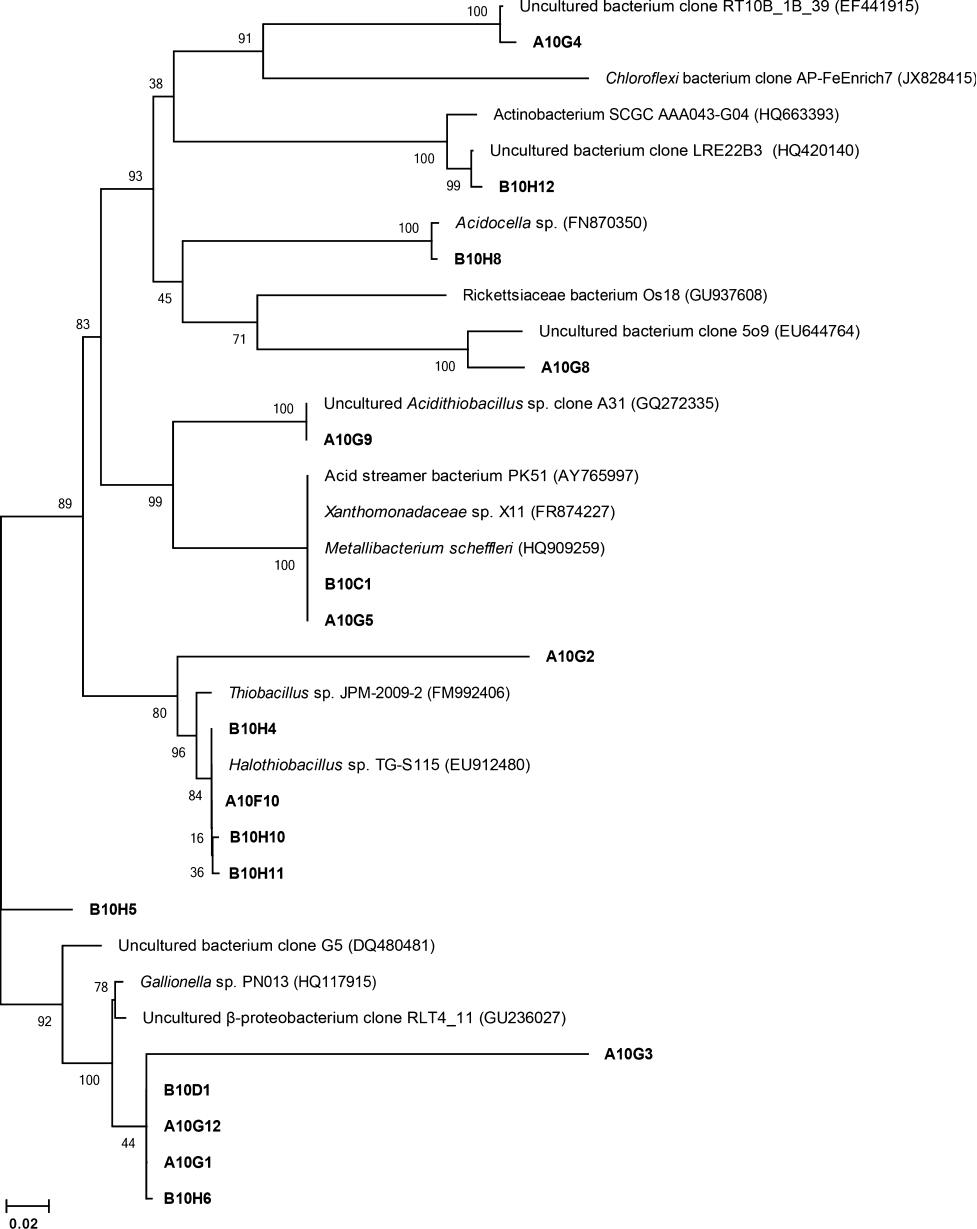
Unrooted neighbor-joining phylogenetic tree of clones from the leaching of the partially oxidized soil fraction inoculated with microorganisms enriched from the Risöfladan experimental field. RFLP clones from this study are shown in bold and bootstrap values are given (100 cycles). Scale bar denotes 2% divergence.

The species present after leaching of the partially oxidized soil fraction at an initial pH of 4.2 were also identified by RFLP analysis (Fig. 3 and Table S1). 47% of the 16S rRNA gene sequences were most similar to clones identified from environments affected by heavy metals and/or low pH. These included A10G1, A10G3, A10G12, B10H6, and B10D1 that were all most similar (85–97%) to a clone from a uranium and heavy-metal-contaminated soil (Sitte *et al*., 2010); A10G5 and B10C1 that were most similar to a clone identified from the arsenic rich AMD in Carnoules, France (Delavat *et al*., 2012); and A10G9 that was 99% similar to an *Acidithiobacillus* sp. identified from copper sulfide leaching (NCBI direct submission). Finally, 53% of the clones were between 84% and 99% similar to a *Halothiobacillus* sp. that is salt-tolerant, ISC-oxidizing chemolithoautotroph (Kelly & Wood, 2000).

## Conclusions

The temperature of the boreal, Risöfladan experimental field in Finland reached a maximum of *c*. 14 °C during the summer that was sufficiently high to allow microbial activity in the PASS. The presence of 16S rRNA gene clones with highest sequence similarity to known acidophilic Fe^2+^ and ISC oxidizers in the oxidized ASS suggests that they play a role in the production and release of acidic, metal-containing solutions from ASS. This was supported by the increased generation of acidity and higher iron released from partially oxidized soils during leaching in the presence of enriched microorganisms from the Risöfladan experimental site.

## Supporting Information

**Fig. S1.** Vertical profile of Ostrobothnian acid sulfate soil farmland with the approximate levels (from top to bottom); (i) agricultural soil (0.0–0.4 m), (ii) oxidized material (0.4–1.2 m), (iii) semi-oxidized ‘transition’ zone (1.2–1.8 m); and (iv) unoxidized metal sulfides (>1.8 m) (A); soil from the oxidized zone showing an oxidized face (B); and sampling from the deepest, unoxidized zone (C).

**Fig. S2.** Scanning electron microscope (SEM) image of a pyrite framboid surrounded by clay and silt minerals in the reduced soil layer.

**Fig. S3.** Soil temperature profiles at 60 (•) and 150 cm (**▪**) depths at the Risöfladen sampling area from June 2011 until December 2011.

**Fig. S4.** Neighbor-joining phylogenetic tree of the Risöfladan experimental field plough layer (30 cm below the surface) with RFLP clones (in bold) and bootstrap values (100 cycles).

**Fig. S5.** Neighbor-joining phylogenetic tree of the Risöfladan experimental field oxidized, acidic layer (75 cm below the surface) with RFLP clones (in bold) and bootstrap values (100 cycles).

**Fig. S6.** Neighbor-joining phylogenetic tree of the Risöfladan experimental field mixed partially oxidized pH 4–6 layer (127 cm) with RFLP clones (in bold) and bootstrap values (100 cycles).

**Fig. S7.** Neighbor-joining phylogenetic tree of the Risöfladan experimental field dark reduced zone (>180 cm) with RFLP clones (in bold) and bootstrap values (100 cycles).

**Fig. S8.** Neighbor-joining phylogenetic tree of enrichment cultures (pH 3) inoculated with soil from the Risöfladan experimental field incubated with ferrous iron (clones A4), tetrathionate (clones B4), and yeast extract (clones C4) as growth substrate.

**Table S1.** 16S RNA gene clones from the Risöfladen field site plough layer (clones V1; 30 cm below the surface), the red oxidized, acidic (V2; 75 cm), the mixed partially oxidized pH 4–6 (V3; 127 cm), and the dark reduced zones (V4; > 180 cm); the enrichment cultures on 50 mM Fe^2+^ (A4), 5 mM tetrathionate (B4), and 0.02% (wt/vol) yeast extract (C4); and duplicate bioleaching of partially oxidized soils (A10 and B10).
