# Supplementary material for: Microbial community potentially responsible for acid and metal release from an Ostrobothnian acid sulfate soil
Source: FEMS Microbiol Ecol. 2013 Feb 26;84(3):555–63. doi: 10.1111/1574-6941.12084 (PMC3732381; doi:10.1111/1574-6941.12084)

**Microbial Community Potentially Responsible for Acid and Metal Release**

**From an Ostrobothnian Acid Sulfate Soil**

Xiaofen Wu^1^, Zhen Lim Wong^1^, Pekka Sten^2^, Sten Engblom^3^,

Peter Österholm^4^ & Mark Dopson^1^

^1^Centre for Ecology and Evolution in Microbial Model Systems (EEMiS), School of Natural Sciences, Linnaeus University, Kalmar, Sweden;

^2^Vaasa University of Applied Sciences, Vaasa, Finland;

^3^Novia University of Applied Sciences, Vaasa, Finland;

^4^Department of Geology and Mineralogy, Åbo Akademi University, Åbo, Finland

**Supplemental Material**

**Supplemental Table 1.** 16S RNA gene clones from the Risöfladen field site plough layer (clones V1; 30cm below the surface), the red oxidized, acidic (V2; 75 cm), the mixed partially oxidized pH 4 to 6 (V3; 127 cm), and the dark reduced zones (V4; >180 cm); the enrichment cultures on 50 mM Fe^2+^ (A4), 5 mM tetrathionate (B4), and 0.02% (wt/vol) yeast extract (C4); and duplicate bioleaching of partially oxidized soils (A10 and B10).

| Sample name | Most similar species in NCBI | Notes | Similarity | Sequence length | Same pattern / total clones | Accession number | |
| --- | --- | --- | --- | --- | --- | --- | --- |
| **V1: 30 cm; V2: 75 cm; V3: 127 cm; V4: >180 cm** | | | | | | |  |
| V1C9 | Uncultured clone 2B_04F (JX098355 ) | Mineral soils | 99% | 492 | 2/12 | JX869453 | |
| V1G9 | Uncultured *Acidimicrobiaceae* clone (HQ730644) | Extreme acid environment | 98% | 484 | 1/12 | JX869457 | |
| V1A10 | Uncultured bacterium clone Carn-cDNA_31 (FR846984) | Sediment from acid mine drainage | 92% | 450 | 2/12 | JX869451 | |
| V1H10 | Uncultured Rubrobacteridae clone EB1123 (AY395442) | Pasture soil | 93% | 490 | 1/12 | JX869458 | |
| V1D11 | Uncultured *Acidimicrobiaceae* clone (HQ730638) | Acidic environment with heavy metals | 100% | 374 | 2/12 | JX869454 | |
| V1F11 | Uncultured bacterium clone Carn-cDNA_41 (FR846993) | Sediment from acid mine drainage | 99% | 449 | 1/12 | JX869456 | |
| V1A12 | *Acidithiobacillus ferrooxidans* strain SS3-P1 (FN686788) | Mining area | 99% | 490 | 1/12 | JX869452 | |
| V1P14 | Uncultured *Bdellovibrio* sp. clone RUGL1-297 (GQ421053) | Roopkund glacier | 98% | 491 | 1/12 | JX869459 | |
| V1E11 | Uncultured bacterium clone 1-b284 (HM065648) | Glacier forefield | 94% | 500 | 1/12 | JX869455 | |
|  |  |  |  |  |  |  | |
| V2A2 | Uncultured bacterium clone DCK-I-19 (DQ660880) | Acid mine drainage | 98% | 453 | 3/25 | JX869460 | |
| V2A3 | Uncultured bacterium clone DCK-I-19 (DQ660880) | Acid mine drainage | 95% | 477 | 2/25 | JX869461 | |
| V2A4 | Uncultured *Firmicutes* clone (EF662688) | Soil | 98% | 530 | 1/25 | JX869462 | |
| V2A5 | Uncultured *Acetobacteraceae* clone B4_86 (AM940750) | Arctic soil | 99% | 494 | 4/25 | JX869463 | |
| V2A8 | Uncultured bacterium clone DCK-I-19 (DQ660880) | Acid mine drainage | 99% | 550 | 3/25 | JX869464 | |
| V2A11 | Uncultured bacterium clone CG224 (JF440441) | Forest soil | 98% | 519 | 1/25 | JX869465 | |
| V2B2 | Uncultured bacterium clone mus-c24 (JN023844) | Temperate highland grassland | 98% | 485 | 1/25 | JX869466 | |
| V2B5 | Uncultured bacterium clone O218406E05 (FN866144) | Rio Tinto acid mine drainage | 99% | 392 | 2/25 | JX869468 | |
| V2B12 | Uncultured *Acidobacteria* clone T15.0004 (EF457347) | Uranium contaminated sediment | 98% | 408 | 1/25 | JX869470 | |
| V2C3 | Uncultured clone 128 (HE603995) | Acidic lignite mine | 99% | 530 | 1/25 | JX869472 | |
| V2B3 | Uncultured actinobacterium clone WaA_G12 (FJ661187) | Soil | 98% | 531 | 2/25 | JX869467 | |
| V2B9 | Uncultured bacterium clone CG224 (JF440441) | Forest soil | 98% | 518 | 1/25 | JX869469 | |
| V2C2 | Uncultured bacterium clone DCK-I-19 (DQ660880) | Acid mine drainage | 99% | 549 | 1/25 | JX869471 | |
| V2C4 | Uncultured bacterium clone DCK-I-19 (DQ660880) | Acid mine drainage | 99% | 494 | 1/25 | JX869473 | |
| V2P37 | Uncultured *Chloroflexi* clone F07_WMSP1 (DQ450737) | Alpine tundra wet meadow soil | 100% | 350 | 1/25 | JX869474 | |
|  |  |  |  |  |  |  | |
| V3C6 | Uncultured bacterium clone 4D2_cons (EF688193) | Anaerobic wastewater treatment | 98% | 541 | 4/9 | JX869475 | |
| V3D2 | Uncultured organism clone SBXZ_5123 (JN436287) | Guerrero Negro Hypersaline Mat | 92% | 557 | 1/9 | JX869476 | |
| V3D5 | Uncultured bacterium clone 4D2_cons (EF688193) | Anaerobic wastewater treatment | 97% | 504 | 3/9 | JX869477 | |
| V3D11 | Uncultured *Firmicutes* bacterium clone B95-6A (FJ609976) | Anoxic estuarine sediment | 99% | 500 | 1/9 | JX869478 | |
|  |  |  |  |  |  |  | |
| V4G1 | Uncultured clone (AB704727) | 250 m depth borehole | 99% | 488 | 1/28 | JX869483 | |
| V4H1 | Uncultured *Dethiobacter* sp. clone L6B-341 (GU000253) | Lake sediment | 99% | 556 | 1/28 | JX869485 | |
| V4E2 | Uncultured bacterium clone Napoli-2B-07 (AY592634) | Sediment | 98% | 490 | 3/28 | JX869481 | |
| V4G2 | Uncultured bacterium clone SCS_HX21_70 (HM598176) | Sediment | 91% | 529 | 1/28 | JX869484 | |
| V4B3 | Uncultured clone GUAY_37enr_Bac82 (FR682630) | Hydrothermal sediments | 94% | 476 | 14/28 | JX869480 | |
| V4F1 | Uncultured *Dethiobacter* sp. clone L6B-341 (GU000253) | Lake sediment | 99% | 555 | 1/28 | JX869482 | |
| V4A2 | Uncultured bacterium clone: KM22B-15 (AB300128) | Holocene mud sediment | 97% | 530 | 1/28 | JX869479 | |
| V4H2 | Uncultured bacterium clone NSED2_132 (JF495356) | Sediment from anoxic fjord | 99% | 546 | 6/28 | JX869486 | |
|  |  |  |  |  |  |  | |
| **Enrichment cultures on Fe^2+^ (A4), tetrathionate (B4), and yeast extract (C4)** | | | | | | | |
| A4E3 | Acid streamer bacterium PK51 (AY765997) | Acid mine drainage from copper mine | 99% | 490 | 6/33 | JX869406 | |
| A4E8 | *Xanthomonadaceae* sp. X11 (FR874227 | Arsenic-rich acid mine drainage | 99% | 490 | 6/33 | JX869409 | |
| A4E9 | *Acidithiobacillus ferrivorans* (FN870327) | Acidic coal mine | 100% | 487 | 3/33 | JX869410 | |
| A4F6 | *Actinobacterium* BGR 105 (GU168008) | Sulfidic Mine Waste Dumps | 99% | 494 | 1/33 | JX869415 | |
| A4F9 | *Xanthomonadaceae* sp. X11 (FR874227) | Arsenic-rich acid mine drainage | 99% | 491 | 1/33 | JX869417 | |
| A4E4 | Uncultured *Acidithiobacillus* sp. DBS1 (JN866827) | Soil near waste copper mineral | 95% | 487 | 1/33 | JX869407 | |
| A4E6 | Uncultured *Sulfobacillus* sp. clone T13 (HM027483) | Bioleaching heap from circuit boards | 97% | 494 | 1/33 | JX869408 | |
| A4E11 | Uncultured bacterium clone 1200m_z_e6 (HM745459) | Acid mine effluent | 99% | 492 | 2/33 | JX869411 | |
| A4F2 | *Acidithiobacillus ferrooxidans* (DQ676506) | Diverse habitats in China | 99% | 491 | 3/33 | JX869412 | |
| A4F3 | Uncultured bacterium clone RT9-ant04-c09-S (JF737865) | Rio Tinto acidic environment | 97% | 446 | 3/33 | JX869413 | |
| A4F5 | Uncultured clone: OY04C2-062 (AB552249) | Volcanic ash deposit | 98% | 459 | 5/33 | JX869414 | |
| A4F7 | Uncultured bacterium clone SCP131 (HQ264763) | Forest soil | 94% | 486 | 1/33 | JX869416 | |
|  |  |  |  |  |  |  | |
| B4F3 | *Xanthomonadaceae* sp. X11 (FR874227) | Arsenic-rich acid mine drainage | 99% | 494 | 4/28 | JX869431 | |
| B4H3 | Uncultured bacterium clone RT8-ant03-e05-W (JF807636) | Rio Tinto acidic environment | 99% | 498 | 1/28 | JX869432 | |
| B4C4 | *Xanthomonadacea*e sp. X11 (FR874227) | Arsenic-rich acid mine drainage | 98% | 498 | 1/28 | JX869428 | |
| B4E4 | *Xanthomonadaceae* sp. X11 (FR874227) | Arsenic-rich acid mine drainage | 97% | 489 | 1/28 | JX869430 | |
| B4B5 | Uncultured bacterium clone: OY05-C122 (AB552135) | Volcanic ash deposit | 97% | 489 | 4/28 | JX869426 | |
| B4C3 | *Xanthomonadaceae* sp. X11 (FR874227) | Arsenic-rich acid mine drainage | 96% | 492 | 16/28 | JX869427 | |
| B4D4 | Uncultured bacterium clone fb11 (DQ303258 ) | Extreme acidic environment, Rio Tinto | 90% | 491 | 1/28 | JX869429 | |
|  |  |  |  |  |  |  | |
| C4B6 | *Thiomonas* sp. FB-Cd (JN885795) | Sediment from uranium-mining area | 99% | 485 | 1/17 | JX869443 | |
| C4C6 | *Thiomonas* sp. FB-Cd (JN885795) | Sediment from uranium-mining area | 99% | 489 | 1/17 | JX869445 | |
| C4G7 | Uncultured bacterium clone: 07Nov8-54 (AB618386) | Upflow Anaerobic Sludge Bed reactor | 94% | 491 | 1/17 | JX869448 | |
| C4H7 | Uncultured bacterium gene clone: OY05-C010 (AB552036) | Volcanic ash deposit | 99% | 498 | 1/17 | JX869450 | |
| C4A8 | Uncultured bacterium isolate DGGE gel band 9 (GQ502921) | Shale cliffs, north-east England | 99% | 483 | 4/17 | JX869442 | |
| C4B8 | *Propionibacteriaceae* sp. H7p (FR874241) | Arsenic-rich acid mine drainage | 99% | 498 | 1/17 | JX869444 | |
| C4G8 | Uncultured bacterium clone RGJ12 (DQ336036) | Copper mine tailings | 90% | 315 | 1/17 | JX869449 | |
| C4G6 | Uncultured bacterium clone SSL_071 (HQ629503) | Acidified forests soil | 98% | 490 | 2/17 | JX869447 | |
| C4C8 | *Xanthomonadaceae* sp. X11 (FR874227) | Arsenic-rich acid mine drainage | 99% | 489 | 5/17 | JX869446 | |
|  |  |  |  |  |  |  | |
| **Duplicate bioleaching of partially oxidized soils (A10 and B10)** | | | | | | | |
| A10F10 | *Halothiobacillus* sp. TG-S115 (EU912480) | Rhizosphere soil | 99% | 490 | 13/23 | JX869418 | |
| A10G1 | Uncultured β-proteobacterium clone RLT4_11 (GU236027) | Heavy metal-contaminated soil | 97% | 499 | 2/23 | JX869419 | |
| A10G2 | *Halothiobacillus* sp. TG-S115 (EU912480) | Rhizosphere soil | 84% | 410 | 1/23 | JX869420 | |
| A10G3 | Uncultured β-proteobacterium clone RLT4_11 (GU236027) | Heavy metal-contaminated soil | 85% | 500 | 1/23 | JX869421 | |
| A10G4 | Uncultured bacterium clone RT10B_1B_39 (EF441915) | Acidic Rio Tinto basin environment | 99% | 493 | 2/23 | JX869422 | |
| A10G5 | *Xanthomonadaceae* sp. X11 (FR874227 | Arsenic-rich acid mine drainage | 99% | 490 | 1/23 | JX869423 | |
| A10G8 | Uncultured bacterium clone 5o9 (EU644764) | Soap lake | 93% | 468 | 1/23 | JX869424 | |
| A10G9 | Uncultured *Acidithiobacillus* sp. clone A31 (GQ272335) | Bioleaching of copper sulfide ores | 99% | 493 | 1/23 | JX869425 | |
| A10G12 | Uncultured β-proteobacterium clone RLT4_11 (GU236027) | Heavy metal-contaminated soil | 97% | 550 | 1/23 | JX888164 | |
| B10H4 | *Halothiobacillus* sp. TG-S115 (EU912480) | Rhizosphere soil | 99% | 488 | 17/39 | JX869435 | |
| B10H5 | Uncultured bacterium clone G5 (DQ480481) | Acid mine drainages (sulfate mine) | 95% | 490 | 1/39 | JX869436 | |
| B10H6 | Uncultured β-proteobacterium clone RLT4_11 (GU236027) | Heavy metal-contaminated soil | 97% | 490 | 9/39 | JX869437 | |
| B10H8 | *Acidocella* sp. (FN870350) | Fe-cycling bacteria in acidic sediments | 99% | 422 | 2/39 | JX869438 | |
| B10H10 | *Halothiobacillus* sp. TG-S115 (EU912480) | Rhizosphere soil | 99% | 437 | 1/39 | JX869439 | |
| B10H11 | *Halothiobacillus* sp. TG-S115 (EU912480) | Rhizosphere soil | 99% | 483 | 1/39 | JX869440 | |
| B10H12 | Uncultured bacterium clone LRE22B3 (HQ420140) | Acidic coal mine drainage | 99% | 485 | 3/39 | JX869441 | |
| B10C1 | *Xanthomonadaceae* sp. X11 (FR874227) | Arsenic-rich acid mine drainage | 99% | 490 | 1/39 | JX869433 | |
| B10D1 | Uncultured β-proteobacterium clone RLT4_11 (GU236027) | Heavy metal-contaminated soil | 97% | 492 | 4/39 | JX869434 | |

**Supplemental Fig. 1.** Vertical profile of Ostrobothnian acid sulfate soil farmland with the approximate levels (from top to bottom); i. agricultural soil (0.0 - 0.4 m), ii. oxidized material (0.4 - 1.2 m), iii. semi-oxidized “transition” zone (1.2 - 1.8 m), and iv. un-oxidized metal sulfides (>1.8 m) (A); soil from the oxidized zone showing an oxidized face (B); and sampling from the deepest, un-oxidized zone (C). Photographs courtesy of Rainer Rosendahl.


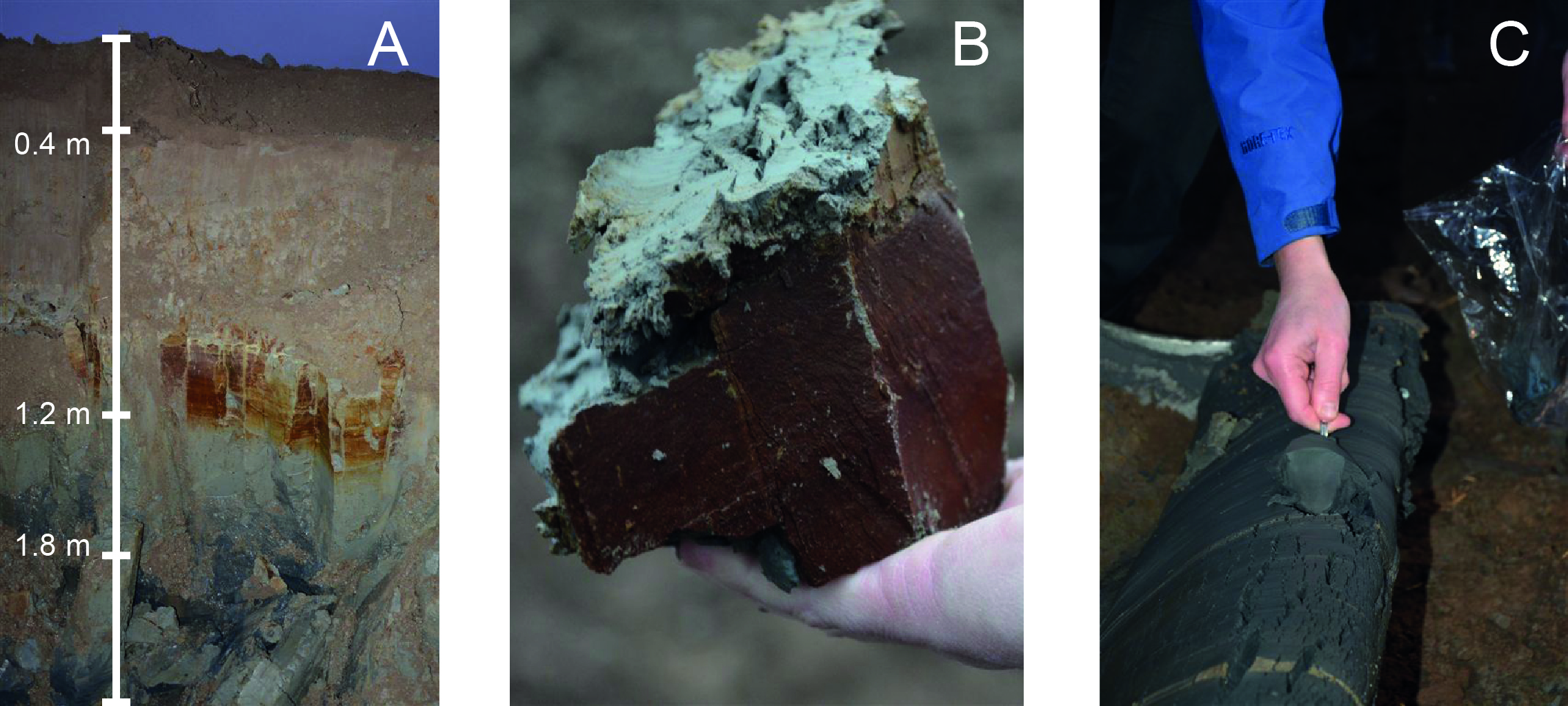


**Supplemental Fig. 2.** Scanning electron microscope (SEM) image of a pyrite framboid surrounded by clay and silt minerals in the reduced soil layer. Scale bar designates 1 µM.


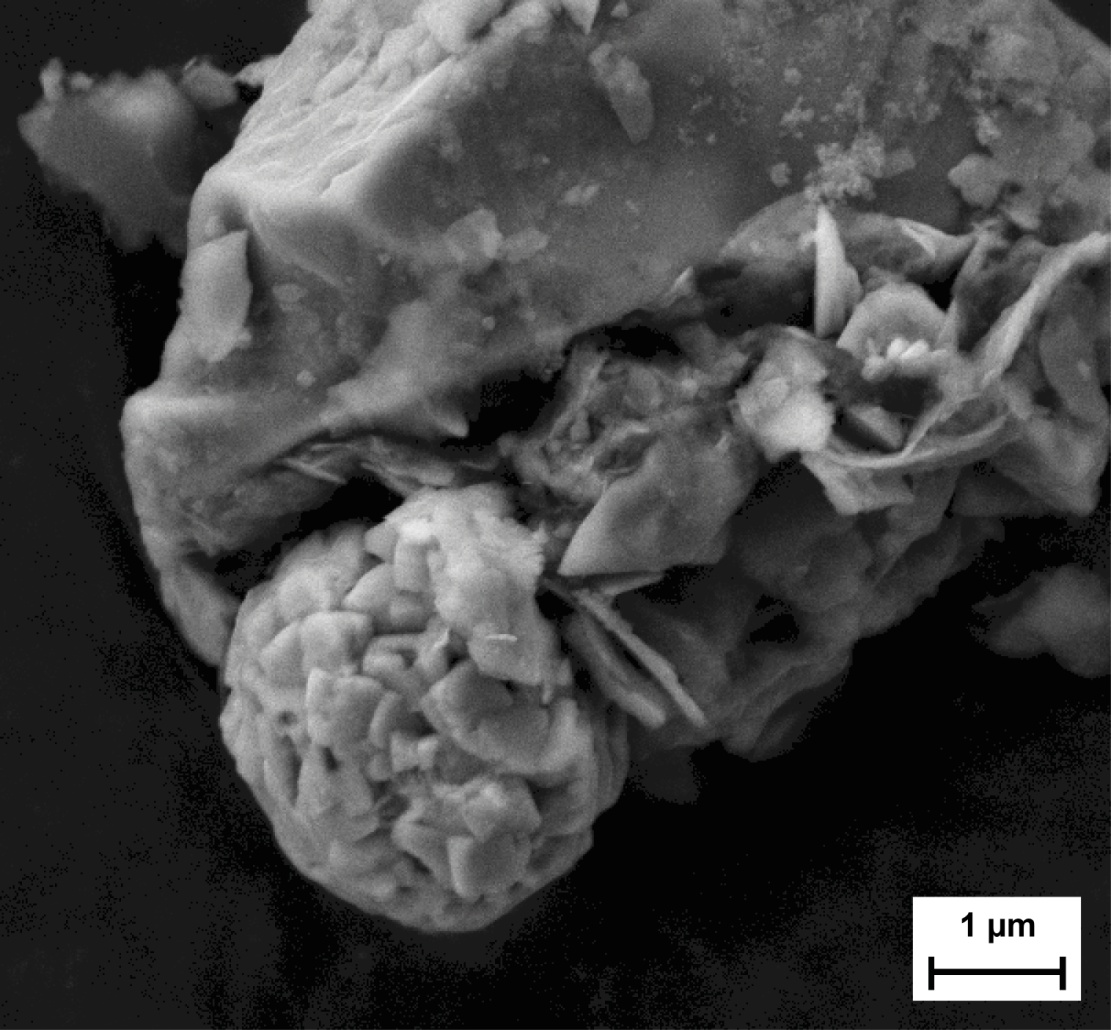


**Supplemental Fig. 3.** Soil temperature profiles at 60 (●) and 150 cm (**■**) depths at the Risöfladen sampling area from June 2011 until December 2011.


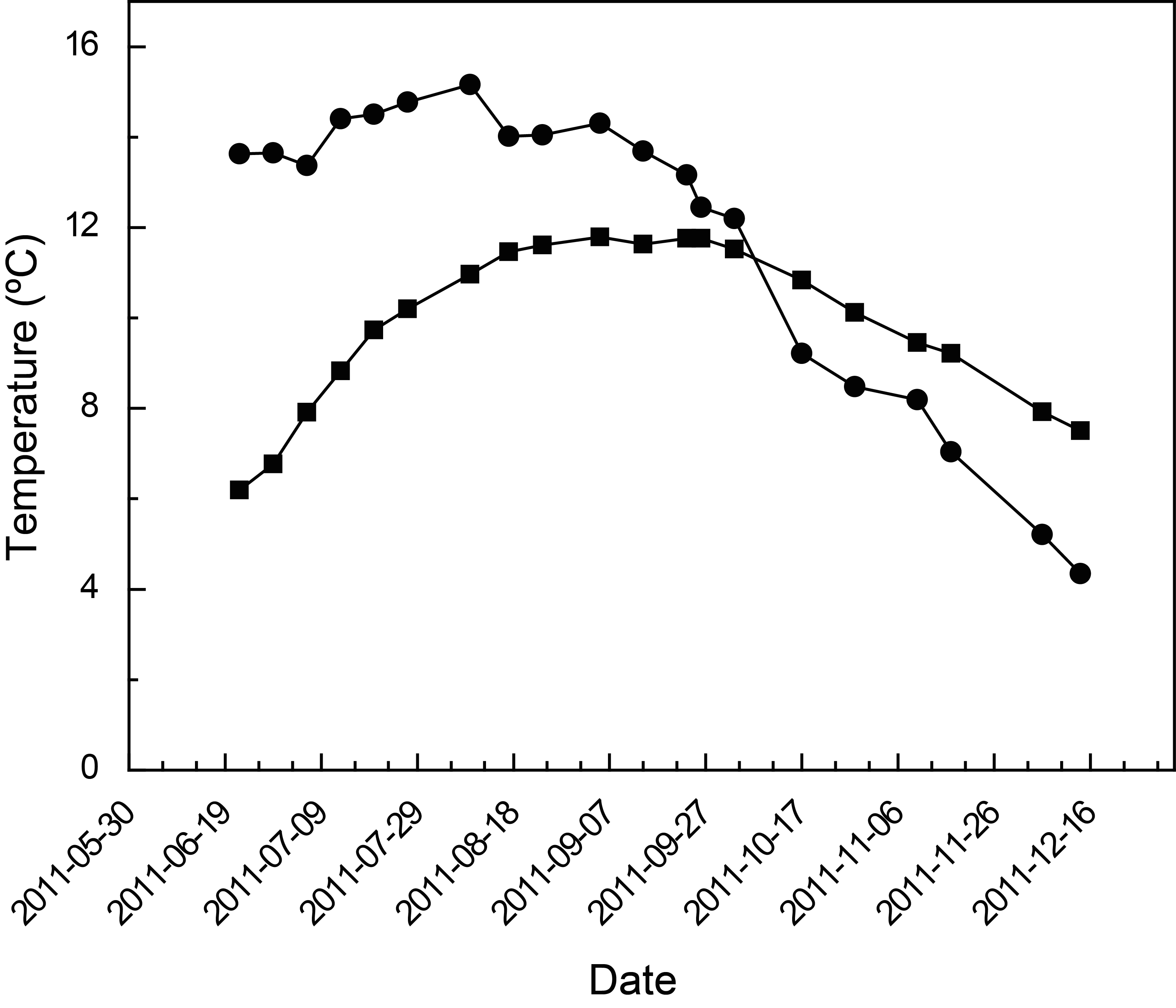


**Supplemental Fig. 4.** Neighbor joining phylogenetic tree of the Risöfladan experimental field plough layer (30 cm below the surface) with RFLP clones (in bold) and bootstrap values (100 cycles). Scale bar denotes 5% divergence.


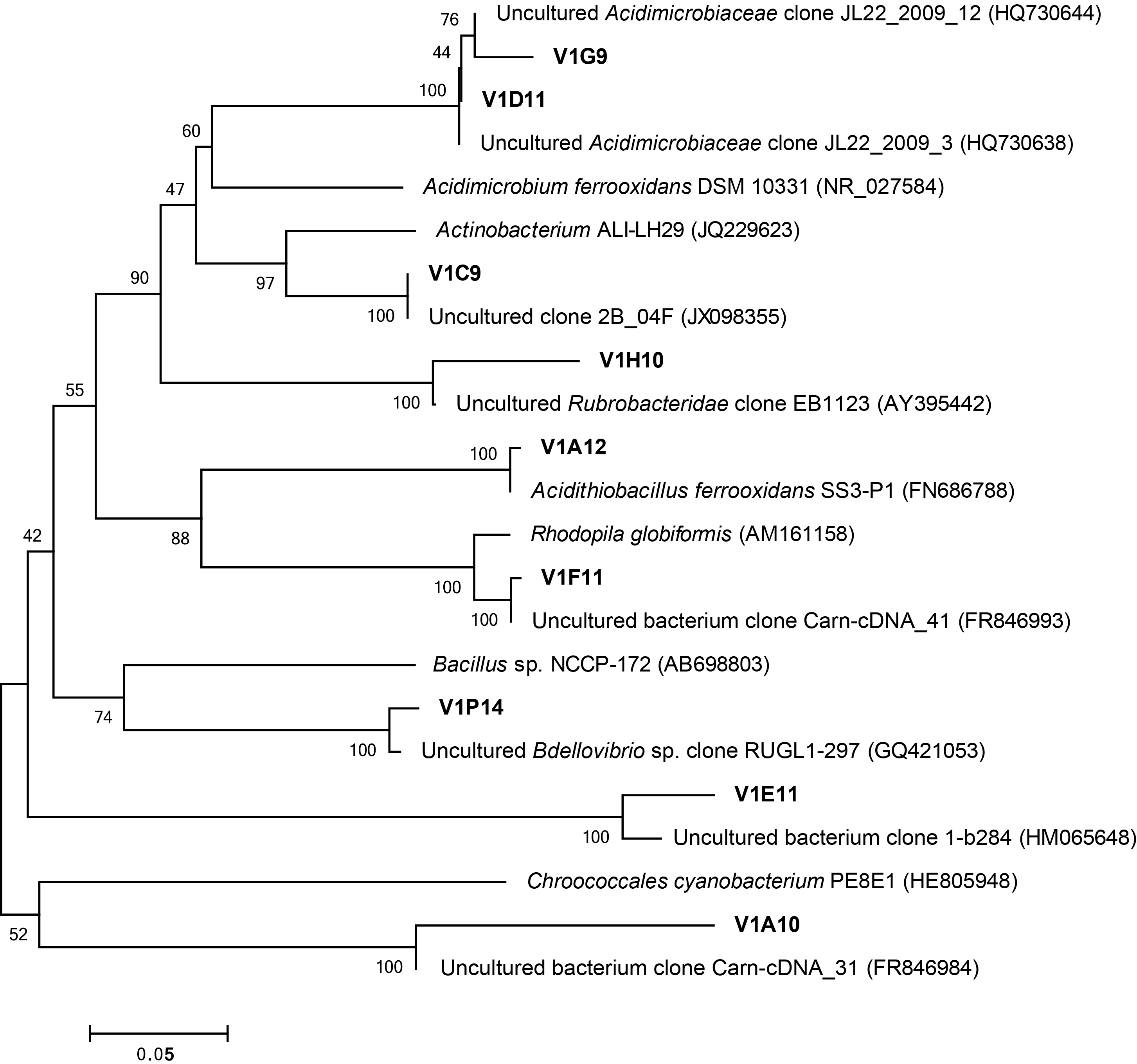


**Supplemental Fig. 5.** Neighbor joining phylogenetic tree of the Risöfladan experimental field oxidized, acidic layer (75 cm below the surface) with RFLP clones (in bold) and bootstrap values (100 cycles). Scale bar denotes 2% divergence.


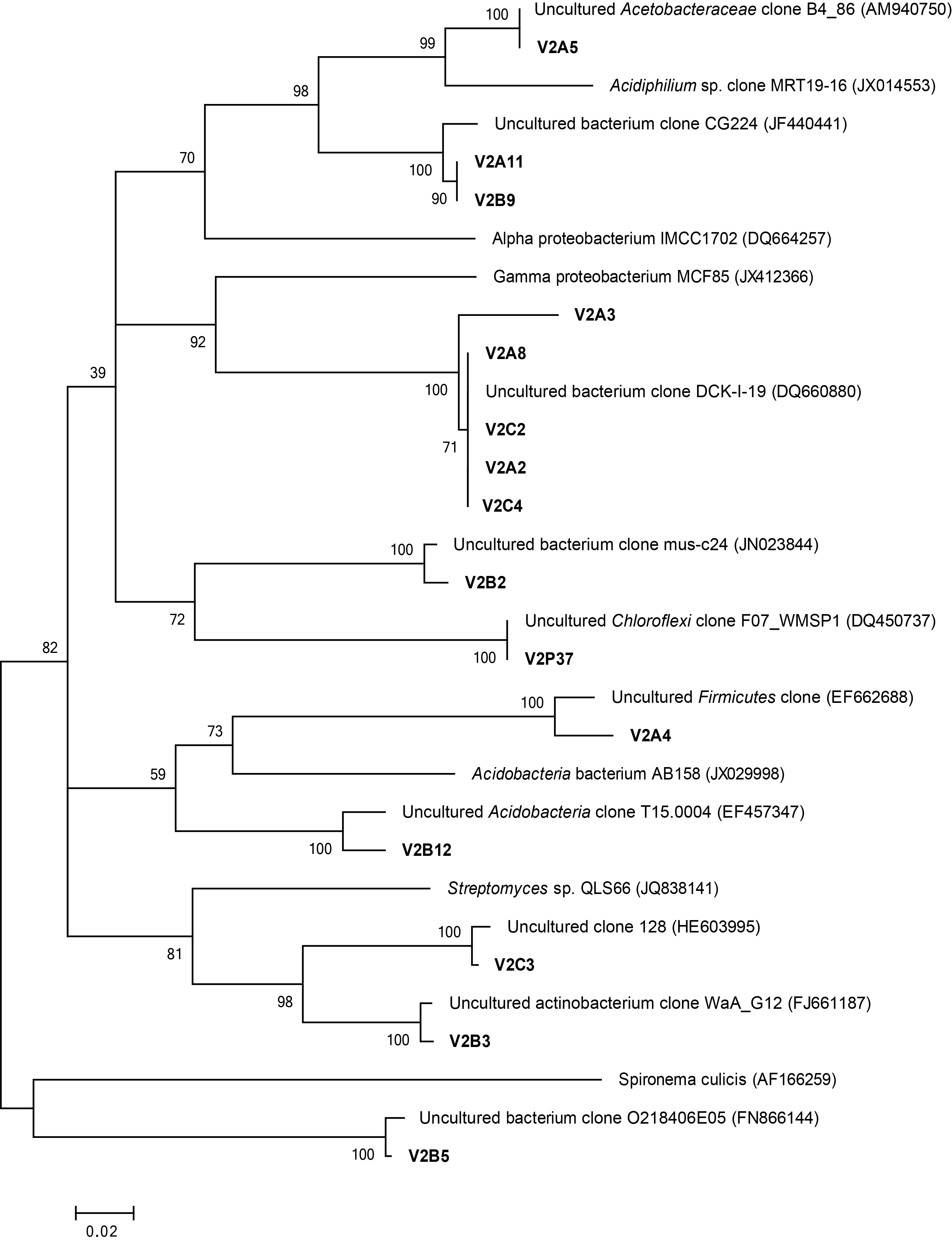


**Supplemental Fig. 6.** Neighbor joining phylogenetic tree of the Risöfladan experimental field mixed partially oxidized pH 4 to 6 layer (127 cm) with RFLP clones (in bold) and bootstrap values (100 cycles). Scale bar denotes 2% divergence.


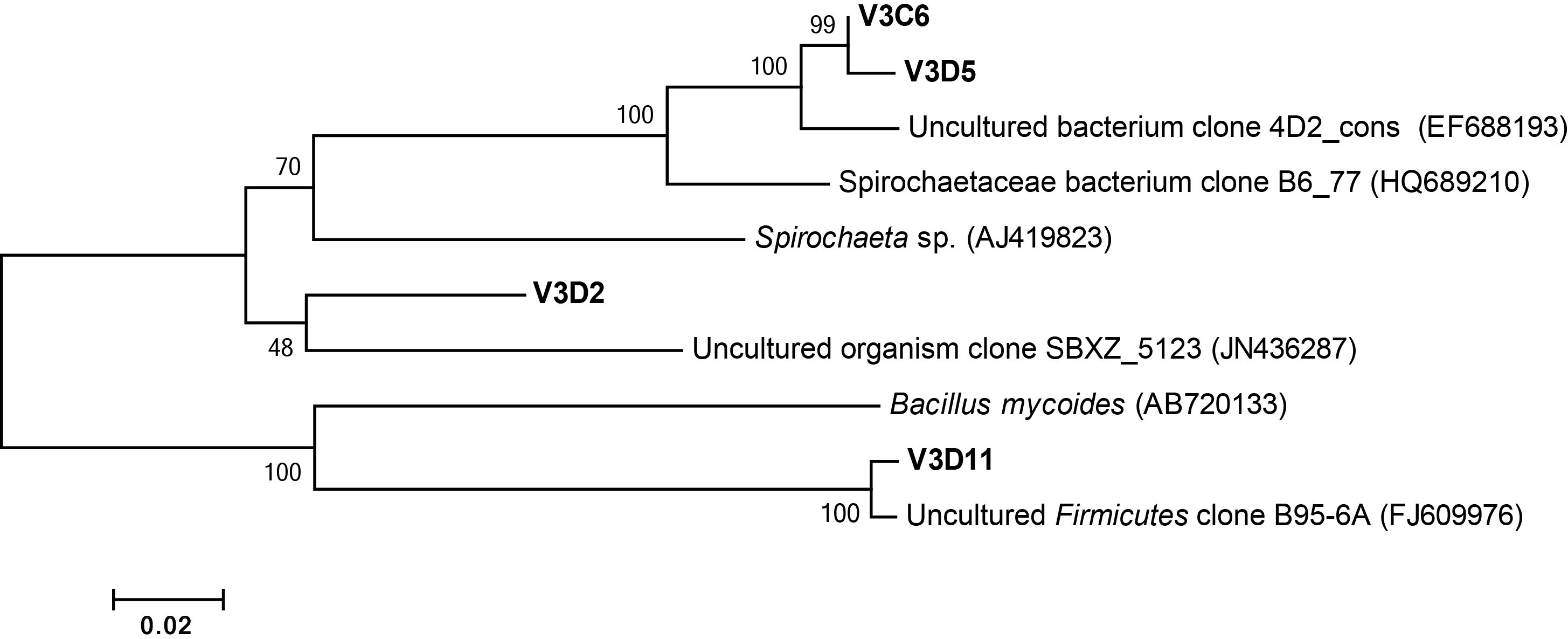


**Supplemental Fig. 7.** Neighbor joining phylogenetic tree of the Risöfladan experimental field dark reduced zone (>180 cm) with RFLP clones (in bold) and bootstrap values (100 cycles). Scale bar denotes 2% divergence.


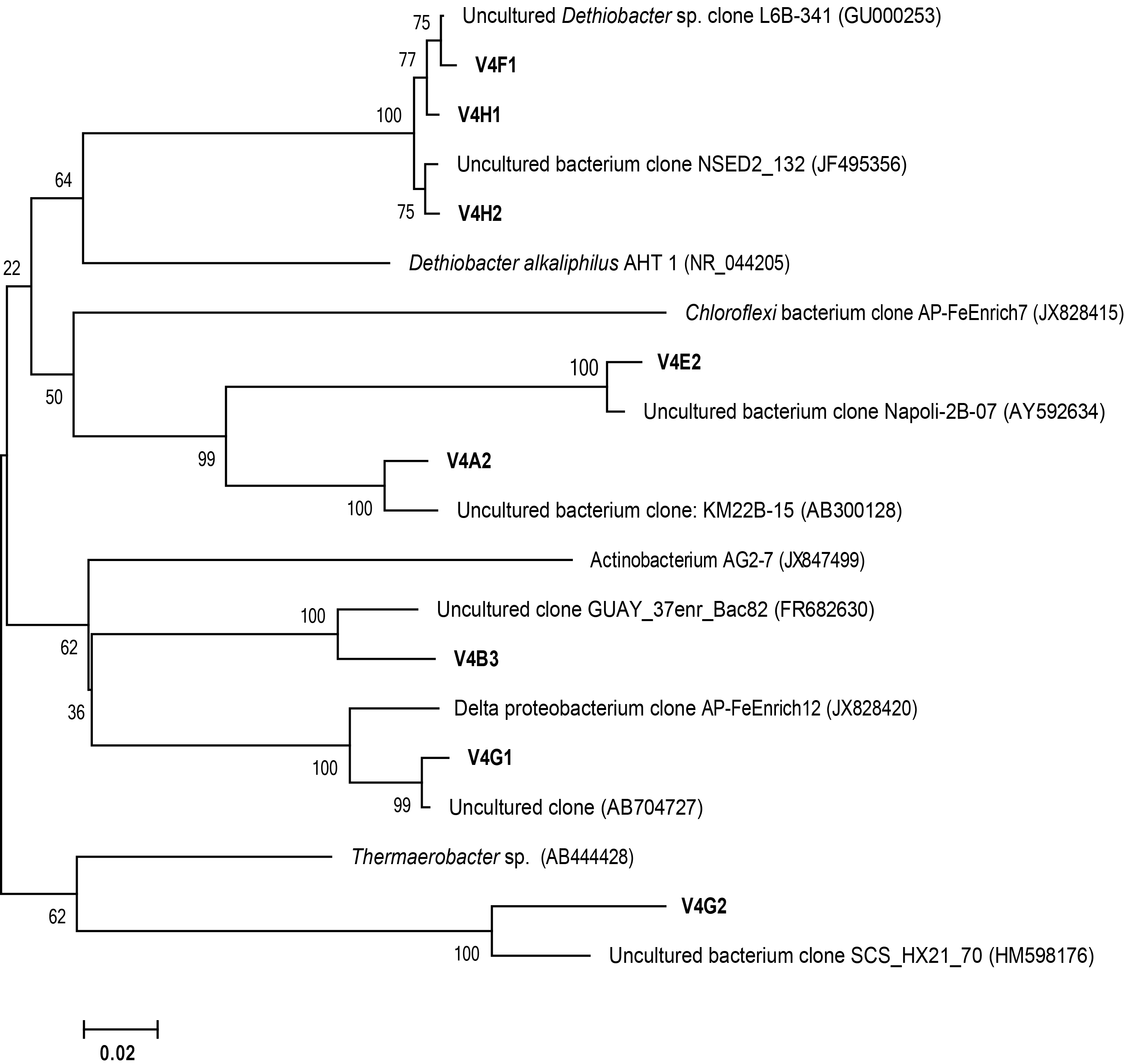


**Supplemental Fig. 8.** Neighbor joining phylogenetic tree of enrichment cultures (pH 3) inoculated with soil from the Risöfladan experimental field incubated with ferrous iron (clones A4), tetrathionate (clones B4), and yeast extract (clones C4) as growth substrate. RFLP clones are in bold and bootstrap values (100 cycles). Scale bar denotes 2% divergence.


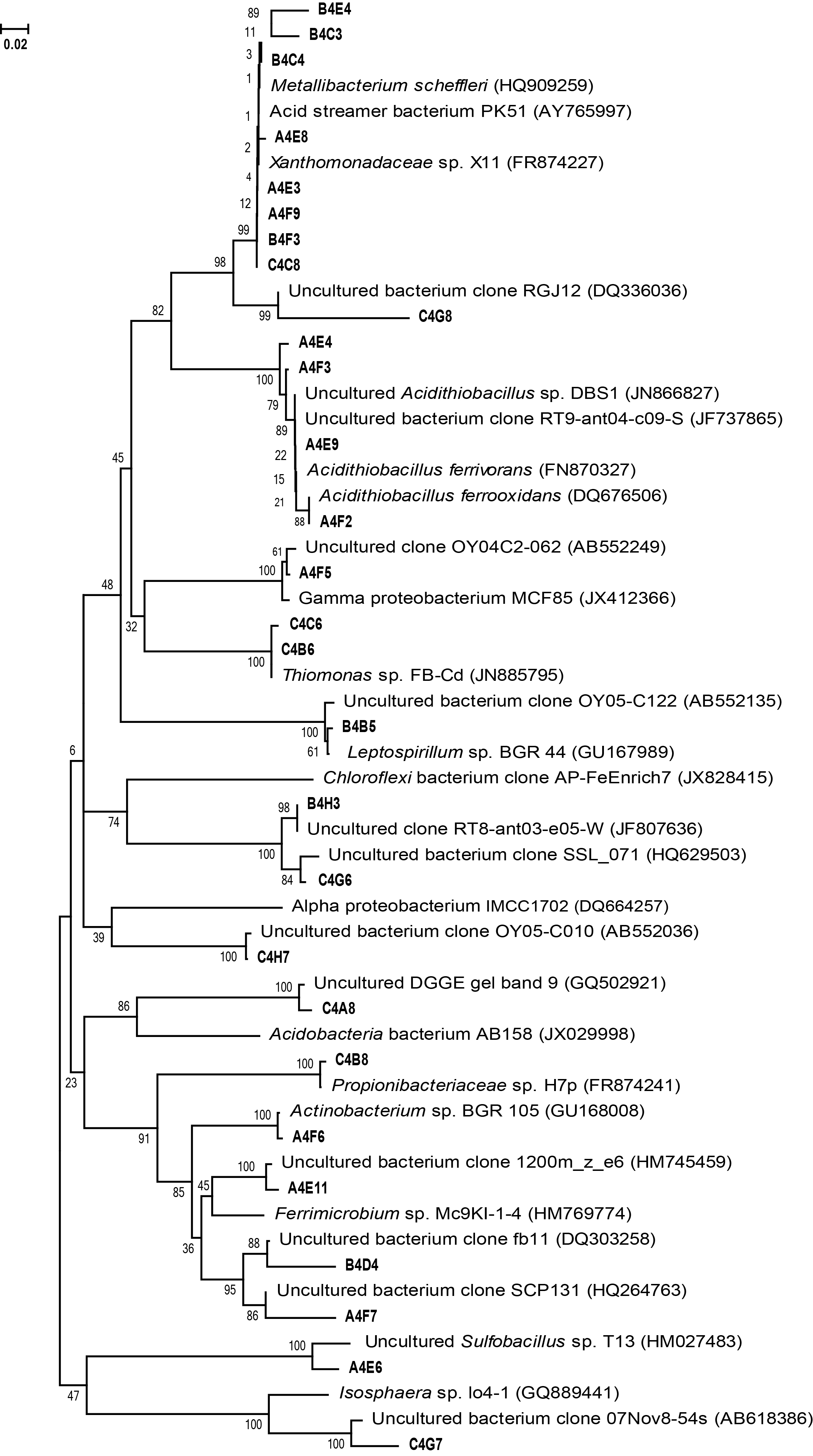

Supplement: Supplementary file 1 [file fem0084-0555-SD1.docx]
